# Delayed discharges in an urban in-patient mental health service in England

**DOI:** 10.1192/pb.bp.113.043083

**Published:** 2014-04

**Authors:** Rob Poole, Alison Pearsall, Tony Ryan

**Affiliations:** 1 Bangor University, Wales, UK; 2 University of Manchester, UK

## Abstract

**Aims and method** To describe the clinical and demographic characteristics of all in-patients experiencing delayed discharge over 3 months in an English urban mental health National Health Service trust. We carried out a cross-sectional case record study with care coordinator questionnaire.

**Results** Overall, 67 in-patients with delayed discharge occupied 18.6% of acute beds. Older in-patients were White, diagnosed with dementia and experienced relatively short admissions. Younger in-patients were often of Black and minority ethnic background with a psychotic diagnosis and long service contact, and sometimes experienced very long admissions. They were similar to a long-stay comparison group. The whole cohort was socially isolated and marginalised, and frequently misused alcohol.

**Clinical implications** People with complex mental health problems can experience long stays in acute care settings. This particularly affects people with psychosis who are isolated in the community. Alcohol misuse is the most common complicating factor. There are insufficient community-oriented rehabilitation services to meet these patients’ diverse needs.

Over the past 25 years there has been an increasing emphasis on the efficient use of in-patient treatment, which is the most expensive form of healthcare. This has drawn attention to patients who are regarded as ‘delayed discharges’ (’bed blockers’ in the older literature). As long ago as 1986, an acute general hospital point prevalence study showed that one in ten in-patients were ‘blocking beds’.^1^ Much of the previous literature relates to services other than acute mental health, but the issue is problematic here, too. This is particularly relevant in the context of increasing concerns about the care of service users who require longer-term in-patient treatment.^2^

In 2002, the Department of Health was urged by the government to produce a clear definition of delayed discharge in mental health services in order to gather better quality information.^3^ This has yet to be achieved. The current Department of Health definition, covering all medical specialties, specifies that the person is medically fit and requires a change of care environment.^4^ A Department of Health report in 2005 suggested that ‘delays in discharge should be considered to start when [such] a care package is delayed. The emphasis should hence be on when a patient is safe for discharge’.^5^ Glasby & Lester^6^ concluded that defining delayed discharge in mental health was complex and varied according to the professional group undertaking the task and their seniority.

At present, in-patients are declared as ‘delayed discharges’ by trusts on the primary criterion that a senior health professional believes that they would be better cared for elsewhere. They are, therefore, a heterogeneous group of people, with varying needs, who are receiving very expensive care but who are not receiving optimal care. They are at risk of institutionalisation and service dependence. This study aims to describe the clinical and demographic characteristics of a group of people regarded as delayed discharge in-patients in an urban mental health service in England.

## Method

The study was based in a specialist mental health trust in a provincial urban area of England. A census or cross-sectional method was used to identify all in-patients who, over a 3-month period, were recorded by the trust to be ‘delayed discharges’ using the 2003 guidance that they were inappropriately placed on the ward and required another care environment.^4^ This was achieved by collecting information on:

- people identified as delayed discharge patients on census day 1 (in October 2009)
- people newly identified as delayed discharge patients on census day 2 (in January 2010)
- people identified as delayed discharge patients but then discharged between the two census dates.

Information was also collected on a comparison group of 12 long-stay patients. These were the 12 in-patients with the longest stays who had not been declared delayed discharge patients by the second census day.

The primary source of information was the trust’s clinical record system, which was largely held electronically. This information was extracted on site by a consultant psychiatrist (R.P.) and a mental health nurse (A.P.) working together. The form that was used is available online (online supplement 1). This sets out the items extracted.

The second source of information was a questionnaire that was sent to the patient’s care programme approach (CPA) coordinator, seeking their views concerning the person’s situation. The coordinator questionnaire is also available online (online supplement 2).

All data were fully anonymised prior to analysis and analysed using SPSS version 20 on Mac OS X.

## Results

### The service

There were 142 acute in-patient beds across 7 wards and 18 beds on 2 psychiatric intensive care units (PICUs). For older adults, there were 4 wards with 77 acute assessment beds. Our study was based on these 237 beds. Older adult continuing care, intellectual disability, substance misuse, alcohol and adolescent beds were excluded. The trust provided 21 rehabilitation beds and third-sector organisations provided 24 rehabilitation beds. These were also excluded from the study.

### Missing data

There was a minimum amount of missing data; for the majority of sociodemographic and clinical variables 100% data collection was achieved. There was one missing data entry for ‘housing status’. For a small number of items, clinical information was unavailable in a few cases. There were no more than four missing entries for each item affected.

The response rate for the CPA coordinator questionnaire was 96.2%.

### Characteristics of the sample

In the 3-month sampling period, 67 people were recorded as delayed discharge patients; 16 of them were under the care of the older adult service. Half of each of the three groups (adult delayed discharges, older adult delayed discharges and long-stay in-patients) were female. The mean age of all delayed discharge patients was 48.1 years (range 20-89). For the long-stay group it was similar (51.8 years, range 26-79).

With regard to ethnicity, there were two Black Caribbean patients aged 50 and 52; all other patients over the age of 50 were White British. It is known that rates of psychosis are high among people of Black and minority ethnic background, and they were over-represented among adult delayed discharges, as would be expected. Their sparse representation among older adult delayed discharges when compared with census data for the area covered by the trust was unexpected, but the number of older adults involved in this study is too low for this finding to be statistically reliable, and the finding requires replication.

Table 1 shows marked differences in length of stay for delayed discharge patients between older adult and general adult services, with longer stays among younger adults. This finding is statistically significant (*P*<0.014, Mann-Whitney test).

There was turnover of delayed discharges during the study period. Of the 43 delayed discharges on census day 1, 9 individuals (21%) had been discharged 3 months later. Of the 24 patients newly listed as delayed discharges between census days 1 and 2, 10 (42%) had been discharged before the second census. At any given time approximately 18.6% of the trust’s acute in-patient beds were occupied by individuals who had been declared as delayed discharges. Among the older adult delayed discharges, turnover tended to be relatively high, most being discharged within a few months. Younger adult delayed discharges, on the other hand, show greater heterogeneity, with a significant minority experiencing very long delays in discharge.

### Diagnosis

Diagnosis was recorded in the clinical record, which normally included ICD-10 code. The largest group in the whole sample had a primary diagnosis of functional psychosis (57%); 11.4% had affective psychosis and 3.8% ‘other psychosis’. The second largest group (15.9%) had a primary diagnosis of dementia. All of the latter were under the care of the older adult service. Of the total sample, 17 (21.5%) had one or more secondary diagnoses. The entire group experienced high levels of disability.

Substance misuse was a common complicating factor in all three groups, affecting 54% of the total sample. Alcohol was the most widely misused substance (Table 2).

### Relationship with the service

All three groups tended to have had lengthy contact with mental health services, although this was most marked among the younger patients. Thirty-three (64.7%) younger delayed discharge patients had been in contact with the service for more than 5 years. The same was true of 10 (83.3%) long-stay patients and 5 (31.3%) older delayed discharge patients.

Of younger delayed discharges, 32 (62.8%) were detained under the Mental Health Act 1983, as were 9 (66.7%) of long-stay patients and 2 (12.5%) of older delayed discharge patients. A high proportion of younger adults were subject to Section 117 of the Act: 86.3% younger adults and 75% of long-stay in-patients compared with 12.5% of older delayed discharge patients.

**Table 1:** Length of stay of delayed discharge patients in the study

Length of admission, days	Adults (*n* = 51)	Older adults (*n* = 16)
Mean	297	174.3
Median	214	124
Minimum	24	70
Maximum	891	681

**Table 2:** Substance misuse

Substance	All cases *n* (%)	DD adults *n* (%)	DD older adults *n* (%)	Long stay *n* (%)
Alcohol	34 (43.0)	24 (47.1)	4 (25.0)	6 (50.0)
Cannabis	28 (35.4)	24 (47.1)	1 (6.3)	3 (25.0)
Amphetamine	9 (11.4)	9 (17.6)		
				
Cocaine	7 (8.9)	7 (13.7)		
				
Opioids	3 (3.8)	3 (5.9)		
				
Benzodiazepine	2 (2.5)	2 (3.9)		
				
MDMA	2 (2.5)	1 (2.0)		1 (8.3)
				
Khat	2 (2.5)	2 (3.9)		
				
Total	79 (100)	51 (100)	16 (100)	12 (100)

DD, delayed discharge; MDMA, 3,4-methylenedioxy-*N*-methylamphetamine.

A majority of younger delayed discharge (*n* = 40, 78.5%) and long-stay (*n* = 10, 83%) patients had been admitted previously, but this was true of only a minority of older delayed discharge patients (*n* = 6, 37.5%). The most recent previous admission tended to have been lengthy, especially for the long-stay patients, and had often ended within a year of readmission.

### Economic and social circumstances prior to admission

Patients tended to live in social housing, with a significant minority having been admitted from residential care. Owner occupation was uncommon (16.5% of all patients), and most frequent among older adults with dementia (43.8%). The second largest housing category was homeless (19% of all cases), although this was confined to the younger patients (24% of this group).

The entire group tended to social isolation, with 5.3% living with a spouse or partner, and 26.6% living with a carer. In 52 cases it was not obvious from the clinical entries which family members, if any, were regularly involved with the patient while they were in hospital.

Only two patients (one each of younger delayed discharge and long-stay patients) had any form of employment immediately prior to admission; 83.5% of the whole group were entirely dependent on state benefits, and this was equally true of all three groups.

### Measures to facilitate discharge

The trust had a specialist discharge planning service, employing staff whose role was to assess need, identify appropriate placements and facilitate funding. Many adult delayed discharges were in contact with this service.

Rates of contact with CPA coordinators during in-patient stays were high, even where the stay was very long. Two-thirds (60.8%) of the whole group had one CPA coordinator during the admission. In 73.5% of cases the CPA coordinator was in contact with the patient at least once every fortnight, and most contacts occurred once a week or more frequently. However, one older adult team with a particularly good record of getting patients out of hospital in a timely way had no contact between CPA coordinators and patients during in-patient stays as a matter of clinical policy. Discharge was coordinated by in-patient staff.

### Reasons for discharge delays

The 12 long-stay patients were excluded from this analysis.

Reasons for delayed discharge were taken from clinical records and from CPA coordinators’ comments.

For 91.0% of the delayed discharge group, the clinical team had determined the type of placement or care package that would be necessary or desirable in order to discharge the patient. In 23% of cases, no suitable placement had been found. In 31.6% of cases, a suitable facility had been identified but no bed was available, and in 10.1% of cases a funding decision was awaited. In 6.4% of cases, assessment for placement was underway (including trial leave) and in 7.6% of cases some element of a care package to support the person in their own home was not yet in place.

Further, 7.6% of patients were waiting for beds in secure facilities. In some cases, patients had been accepted for transfer to a low secure unit, but then experienced a long wait on an acute ward. When a bed became available they were no longer deemed to be in need of moving to a low secure unit, but were not fit for discharge into the community. Other specific needs created similar problems, for example, combined psychosis and substance misuse problems. There were a handful of people with intractable and apparently irresolvable problems, for example, failed asylum seekers with no right to funding.

## Discussion

Our study suggests that the problem of delayed discharge was not improving in 2009-2010. In 2006, the reported rate of delayed discharges in English mental health trusts was 9%.^7^ Although differences in definition dictate caution in comparisons, a finding of 18.6% in our study is unlikely to have arisen from differing definitions alone. The regulator for National Health Service (NHS) foundation trusts sets among its standards for mental health foundation trusts a target of not more than 7.5% of people occupying hospital beds as delayed discharges.^8^

In our study, delayed discharge was a greater problem in services for younger adults than in older adult services. Previous studies have shown high rates of delayed discharges among older adult services,^9,10^ learning disability services^11^ and brain injury services.^12^ As far as we are aware, ours is the first study to directly compare delayed discharges in younger and older person’s services in a single trust. A finding of longer delays in discharge among younger people is of particular concern, as working-age adults are the largest group of mental health service users.

In our study, the long-stay and adult delayed discharges were similar in clinical diagnosis, history of contact with services and demographic features. There were differences with the older adult delayed discharge group. These appear to be a consequence of the much higher level of organic cerebral disease in that group. Overall, the entire sample was quite unwell, with high levels of disability. They were also economically disadvantaged and socially isolated. Rates of substance misuse were high, especially alcohol misuse.

The Community Care (Delayed Discharges etc.) Act 2003 requires local authorities to reimburse NHS trusts where beds have been blocked due to lack of community care. Although this penalty does not apply in mental health, there is implication that lack of effort by Social Service departments is a major cause of delayed discharges. A number of papers have challenged this,^13,14^ suggesting that reasons for discharge delays are complex and are related to the wider health economy. Some studies have tried to identify causes for delay. Glasby & Lester^6^ undertook a narrative literature review on delayed discharge from adult mental health services published between 1992 and 2004. Among their findings were that causes included waiting for either more intensive care (secure or specialist provision) or less intensive care (residential care or home-based support). Lack of funding or lack of appropriate services were frequently cited. A 2004 postal survey of all 83 English mental health trusts produced similar findings.^15^ Causes included waiting for assessment, funding problems, waiting for residential, nursing or domiciliary care packages, and patients or their families exercising choice over placement.

These findings are reflected in our study. Waiting for a bed to become available in a specialist facility was common, although there were also problems in identifying placements offering some types of specialist care (e.g. for people with combined mental illness and substance misuse problems). Overall, a lack of appropriate and available placements appeared to be a bigger problem than lack of funding.

The majority of our delayed discharge sample was profoundly social isolated. Victor & Healy^16^ identified the lack of a carer or family member as a key factor causing discharge delays in older adults, and it seems that this association is equally evident in younger people.

Baumann *et al*^17^ found that better-performing services in respect of non-mental health delayed discharges had more efficient internal processes, such as discharge planning nurses, staff with a role to prevent unnecessary admissions and better joint working arrangements between health and social care agencies. The trust in this study had a well-established team of discharge-planning nurses, therefore the high rate of delayed discharges cannot be attributed to poor engagement with the problem.

There was nothing in our findings to suggest that lack of effort among staff contributed to delayed discharges. In general hospital acute care, Petersson *et al*^18^ found that many of the problems which staff struggled to overcome were beyond their influence, yet they continued to devise processes to reduce delayed discharges. We recognised this pattern in our staff group.

The majority of the younger adult delayed discharge group in our study, together with the long-stay group, appeared to be in need of psychiatric rehabilitation. There are few such services available within the NHS.^19,20^ If delayed discharge is becoming more common, one reason may be an increasing reluctance to purchase placements in the private sector owing to financial constraints. Acute mental health wards may now be feeling the effects of pressure to reduce the use of ‘out of area’ placements^21^ and the consequent retreat from the ‘virtual asylum’.^22^

Concern over lack of provision for ‘new long-stay patients’ has been expressed for many years.^23,24^ Previous studies have identified a similar population on acute mental health wards, apparently in need of rehabilitation.^25,26^ There is a substantial financial cost involved in caring for (or what some might describe as warehousing) people inappropriately on acute mental health wards. What is more distressing is the clear evidence that people with these problems can recover in the right environment. Studying people initially assessed as intractably mentally ill, Trieman & Leff^27^ found that, over a 5-year period, 29 (40%) of a group of 72 people previously regarded as unable to live within community settings (such as care and nursing homes) were able to do so through a slow-stream approach to rehabilitation. Our delayed discharge and long-stay populations, on the other hand, showed a tendency to repeated lengthy hospital stays punctuated by relatively brief periods in the community.

The data presented here were collected 3 years ago. There have been three major changes in English mental health services since then. First, there has been a steady increase in the use of community treatment orders (CTOs), which were introduced to prevent ‘revolving-door’ admissions. However, the OCTET study^28^ has convincingly shown that CTOs are no more effective than older patterns of compulsion. Second, there has been significant retraction of mental health services owing to public sector spending restrictions, with reductions in staffing in some locations. It is widely believed that this has worsened the problem, but no data are available at present. Finally, payment by results has been introduced, with a profound change in remuneration for service providers. Again, no data are available on the impact of this, but our impression is that it has had little effect to date; payment by results does not touch on the key problems that we have identified.

## Limitations

Our study has some limitations. The numbers in the older adult delayed discharge and long-stay groups are relatively small. Some problems in discharging patients may have been idiosyncratic to the trust studied. We relied on clinical records and questionnaire responses from CPA coordinators rather than assessing patients ourselves. Nonetheless, there is no reason to suppose that our findings are systematically misleading. There continues to be a group of people with multiple and complex problems who are not well served by mental health services while generating a high cost for unsatisfactory care. They correspond to the population for whom mental health services were established in the first instance. We come to this state of affairs after more than a decade of mainly private provision for people with chronic mental health difficulties,^29^ and more of the same has no credibility as an answer to their problems. What is needed is serious investment in modern, community-oriented rehabilitation services.^30^
